# RNA-seq data and surprisal analysis of *icl* mutant and control strain of the green microalga *Chlamydomonas reinhardtii* during day/night cycles

**DOI:** 10.1016/j.dib.2018.09.104

**Published:** 2018-10-02

**Authors:** R. Willamme, K.A. Bogaert, F. Remacle, C. Remacle

**Affiliations:** a Genetics and Physiology of Microalgae, UR InBios, Chemin de la Vallée, 4, University of Liège, 4000 Liège, Belgium; b Theoretical Physical Chemistry, UR MOLSYS, Allée du 6 Août, 11, University of Liège, 4000 Liège, Belgium

## Abstract

The data presented in this article are associated to the research article “Surprisal analysis of the transcriptomic response of the green microalga *Chlamydomonas* to the addition of acetate during day/night cycles” (Willamme et al., 2018) [1]. Here the RNA-seq data of the *icl* mutant, a null mutant of the isocitrate lyase gene, and its control are summarized and the FPKM values are listed. The data were analysed using surprisal analysis and the genes contributing the strongest to the mutant and wild type phenotype are listed. The raw data are accessible at BioProject PRJNA437393 with SRA accession number SRP136101 (experiments SRX3824204–SRX3824249). The raw data set and expression values used for surprisal analysis are made public to enable critical or extended analyses.

## Specifications table

**Table:** 

Subject area	*Biology*
More specific subject area	*transcriptomic data (RNA-seq) on the microalga Chlamydomonas reinhardtii*
Type of data	*Tables*
How data was acquired	*Next generation sequencing (NGS)*
Data format	*tables (doc files and csv files)*
Experimental factors	*NA*
Experimental features	*Two strains of the microalga* Chlamydomonas *have been submitted to day/night cycles in a cultivation medium containing acetate as organic carbon source and sampled every 4 h for transcriptomic analysis by RNA-seq during 28 h. Three biological replicates of each strain and for each time point have been analysed. One strain is a null mutant of the isocitrate lyase gene (icl) and the other strain is a control strain (iclC). The isocitrate lyase enzyme is essential for the glyoxylate cycle, required for acetate metabolization. Surprisal analysis has been used to understand the transcriptomic response of both strains to the diurnal rhythm and the presence of acetate.*
Data source location	*NA*
Data accessibility	Raw data of RNA seq analysis are available on Sequence Read Archive (SRA) database and connected to bioproject PRJNA437393.
Related research article	Surprisal analysis of the transcriptomic response of the green microalga *Chlamydomonas* to the addition of acetate during day/night cycles in revision

## Value of the data

● FPKM values of the raw data of the transcriptomics analysis performed in the associated research paper are found and allow comparison with other transcriptomic data obtained in the microalga *Chlamydomonas*.
● KEGG pathways used for the surprisal analysis are found and allow comparison with other papers dealing with differential gene expression analyses.
● All the data supplied here are impossible to display in the accompanying research paper because of their size.

## 1 Data

The dataset of this article provides information on raw RNA-seq reads obtained from the *icl* mutant and the control strain over the diurnal cycle. Data shared concerns the reads information for each biological replicate (Data in Brief 1), the FPKM values of the processed data (Data in Brief 2 and 3), the number of KEGG pathways used in the surprisal analysis (Data in Brief 4), the list of genes relevant for the surprisal analysis of the transcriptomics data (Data in Brief 5–7).

List of shared data is found in the Supplementary material.

## 2 Experimental design, materials, and methods

The experimental design has been described in the accompanying research paper (‘Surprisal analysis of the transcriptomic response of the green microalga *Chlamydomonas* to the addition of acetate during day/night cycles’ by Willamme et al. [1]).

## Footnotes

Appendix A Supplementary data associated with this article can be found in the online version at https://doi.org/10.1016/j.dib.2018.09.104.
