# Supplementary material for: RNA-seq data and surprisal analysis of icl mutant and control strain of the green microalga Chlamydomonas reinhardtii during day/night cycles
Source: Data Brief. 2018 Oct 2;21:351–3. doi: 10.1016/j.dib.2018.09.104 (PMC6197743; doi:10.1016/j.dib.2018.09.104)
Supplement: Supplementary file 1 — Supplementary material [file mmc1.pdf]

Confl icts of Interest Statement

Manuscript title: **RNA-seq data and surprisal analysis of *icl* mutant and control strain of the green microalga *Chlamydomonas reinhardtii* during day/night cycles**

The authors whose names are listed immediately below certify that they have NO affi liations with or involvement in any organization or entity with any fi nancial interest (such as honoraria; educational grants; participation in speakers' bureaus; membership, employment, consultancies, stock ownership, or other equity interest; and expert testimony or patent-licensing arrangements), or non-fi nancial interest (such as personal or professional relationships, affi liations, knowledge or beliefs) in the subject matter or materials discussed in this manuscript.

Author names:

Remi Willamme

Kenny Arthur Bogaert,

Françoise Remacle

Claire Remacle

The authors whose names are listed immediately below report the following details of affi liation or involvement in an organization or entity with a fi nancial or non-fi nancial interest in the subject matter or materials discussed in this manuscript. Please specify the nature of the confl ict on a separate sheet of paper if the space below is inadequate.

Author names:

This statement is signed by all the authors to indicate agreement that the above information is true and correct (*a photocopy of this form may be used if there are more than 10 authors*):

Author's name (typed)	Author's signature	Date
Kenny Bogaert	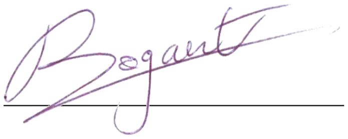	15th of May 2018
