# Supplementary material for: RNA-seq data and surprisal analysis of icl mutant and control strain of the green microalga Chlamydomonas reinhardtii during day/night cycles
Source: Data Brief. 2018 Oct 2;21:351–3. doi: 10.1016/j.dib.2018.09.104 (PMC6197743; doi:10.1016/j.dib.2018.09.104)
Supplement: Supplementary file 2 — Supplementary material [file mmc2.docx]

|  | **Identifier** | **Sample** | **Time** | **Run1** | **Run2** | **Run3** | **Total trimmed reads** | **Uniquely mapped reads** | |
| --- | --- | --- | --- | --- | --- | --- | --- | --- | --- |
| E570 | S1 | icl-1 | 0 | 7,217,147 | 9,986,544 | 8,899,546 | 24,594,592 | 10,889,198 | 44.27% |
| E571 | S2 | icl-2 | 0 | 6,985,359 | 9,593,406 | 8,567,402 | 23,672,579 | 5,397,631 | 22.80% |
| E572 | S3 | icl-3 | 0 | 8,100,615 | 11,034,273 | 9,882,926 | 27,233,161 | 16,728,413 | 61.43% |
| E573 | S4 | icl-1 | 4 | 6,467,075 | 9,146,339 | 8,745,481 | 22,827,602 | 6,414,517 | 28.10% |
| E574 | S5 | icl-2 | 4 | 7,383,804 | 10,553,381 | 10,073,812 | 26,328,898 | 5,172,493 | 19.65% |
| E575 | S6 | icl-3 | 4 | 6,743,101 | 9,431,694 | 9,013,797 | 23,672,337 | 8,634,465 | 36.47% |
| E576 | S7 | icl-1 | 8 | 6,247,151 | 8,500,819 | 7,622,941 | 20,935,847 | 9,267,671 | 44.27% |
| E577 | S8 | icl-2 | 8 | 6,561,665 | 9,096,924 | 8,113,833 | 22,299,466 | 11,949,621 | 53.59% |
| E578 | S9 | icl-3 | 8 | 6,798,083 | 9,320,352 | 8,290,004 | 22,864,576 | 12,424,631 | 54.34% |
| F077 | S10_merged | icl-1 | 12 | 32,755,414 | | | 29,897,157 | 28,340,363 | 94.79% |
| F078 | S11_merged | icl-2 | 12 | 33,261,222 | | | 30,053,211 | 28,237,858 | 93.96% |
| F079 | S12_merged | icl-3 | 12 | 34,400,218 | | | 31,270,607 | 29,713,890 | 95.02% |
| F080 | S13_merged | icl-1 | 16 | 31,882,527 | | | 28,375,109 | 26,673,053 | 94.00% |
| F081 | S14_merged | icl-2 | 16 | 34,901,956 | | | 31,731,913 | 26,074,281 | 82.17% |
| F082 | S15_merged | icl-3 | 16 | 50,662,389 | | | 45,946,899 | 43,403,802 | 94.47% |
| E585 | S16 | icl-1 | 20 | 7,284,467 | 9,977,663 | 8,904,835 | 24,456,403 | 15,934,392 | 65.15% |
| E586 | S17 | icl-2 | 20 | 7,200,127 | 10,004,412 | 8,931,750 | 24,432,043 | 13,946,239 | 57.08% |
| E587 | S18 | icl-3 | 20 | 7,441,326 | 10,105,891 | 9,078,986 | 24,839,544 | 15,642,532 | 62.97% |
| E588 | S19 | icl-1 | 24 | 7,311,700 | 10,128,151 | 8,994,962 | 24,857,333 | 10,220,754 | 41.12% |
| E589 | S20 | icl-2 | 24 | 6,731,836 | 9,379,166 | 8,373,664 | 22,833,919 | 9,439,133 | 41.34% |
| E590 | S21 | icl-3 | 24 | 7,008,238 | 9,563,936 | 8,602,426 | 23,498,155 | 6,177,451 | 26.29% |
| E591 | S22 | icl-1 | 28 | 6,844,960 | 9,360,993 | 8,320,745 | 23,089,569 | 5,775,659 | 25.01% |
| E592 | S23 | icl-2 | 28 | 7,056,772 | 9,826,967 | 8,745,082 | 24,069,072 | 6,828,397 | 28.37% |
| E593 | S24 | icl-3 | 28 | 7,315,756 | 10,170,840 | 9,052,778 | 24,916,730 | 9,455,912 | 37.95% |
| E594 | S25 | iclC-1 | 0 | 5,991,675 | 7,940,724 | 7,155,052 | 19,251,804 | 12,949,495 | 67.26% |
| E595 | S26 | iclC-2 | 0 | 6,839,848 | 9,218,043 | 8,361,172 | 22,856,712 | 17,864,109 | 78.16% |
| E596 | S27 | iclC-3 | 0 | 7,254,089 | 10,014,821 | 9,636,648 | 25,176,694 | 15,836,513 | 62.90% |
| E597 | S28 | iclC-1 | 4 | 6,017,338 | 8,350,869 | 7,473,415 | 20,405,724 | 8,670,588 | 42.49% |
| E598 | S29 | iclC-2 | 4 | 6,546,781 | 9,009,950 | 8,748,220 | 22,560,512 | 9,900,506 | 43.88% |
| E599 | S30 | iclC-3 | 4 | 6,864,268 | 9,557,542 | 9,156,173 | 24,012,380 | 9,293,086 | 38.70% |
| E600 | S31 | iclC-1 | 8 | 7,327,088 | 9,855,745 | 8,902,573 | 24,320,636 | 19,309,180 | 79.39% |
| E601 | S32 | iclC-2 | 8 | 7,256,705 | 10,058,106 | 9,034,513 | 24,560,391 | 20,142,239 | 82.01% |
| E602 | S33 | iclC-3 | 8 | 7,423,799 | 10,226,270 | 9,043,798 | 25,081,167 | 8,072,448 | 32.19% |
| E603 | S34 | iclC-1 | 12 | 6,976,862 | 9,556,081 | 8,469,828 | 23,395,539 | 8,920,152 | 38.13% |
| E604 | S35 | iclC-2 | 12 | 7,780,492 | 10,572,880 | 9,398,663 | 25,931,665 | 19,266,856 | 74.30% |
| E607 | S38 | iclC-2 | 16 | 7,212,304 | 9,907,992 | 8,763,376 | 24,335,774 | 8,009,339 | 32.91% |
| E608 | S39 | iclC-3 | 16 | 7,725,427 | 10,495,602 | 9,337,691 | 25,786,338 | 15,740,171 | 61.04% |
| E609 | S40 | iclC-1 | 20 | 7,425,444 | 10,198,295 | 9,097,592 | 24,989,399 | 19,774,947 | 79.13% |
| E610 | S41 | iclC-2 | 20 | 7,582,576 | 10,550,976 | 9,460,866 | 25,936,035 | 20,327,813 | 78.38% |
| E611 | S42 | iclC-3 | 20 | 7,994,283 | 11,079,058 | 9,964,672 | 27,283,509 | 18,219,098 | 66.78% |
| E612 | S43 | iclC-1 | 24 | 8,570,183 | 11,827,426 | 10,616,979 | 29,080,836 | 26,421,509 | 90.86% |
| E613 | S44 | iclC-2 | 24 | 7,361,546 | 10,242,117 | 9,194,225 | 25,008,708 | 20,489,891 | 81.93% |
| E614 | S45 | iclC-3 | 24 | 7,427,254 | 10,310,636 | 9,284,406 | 25,280,845 | 13,996,128 | 55.36% |
| E615 | S46 | iclC-1 | 28 | 7,541,377 | 10,466,620 | 9,394,935 | 25,707,308 | 14,065,243 | 54.71% |
| E616 | S47 | iclC-2 | 28 | 7,941,287 | 11,178,096 | 9,952,481 | 27,369,675 | 13,964,405 | 51.02% |
| E617 | S48 | iclC-3 | 28 | 7,826,064 | 10,993,591 | 9,816,724 | 26,842,677 | 22,396,041 | 83.43% |

**Data in Brief 1**. Reads information for each sample.

| **KEGG Pathway** | | | **Number of genes in our analysis** | **Total number of genes** | **KEGG Pathway** | | | **Number of genes in our analysis** | **Total number of genes** |
| --- | --- | --- | --- | --- | --- | --- | --- | --- | --- |
| 1 | ko01200 | Carbon metabolism | 129 | 140 | 43 | ko00196 | Photosynthesis - antenna proteins | 23 | 23 |
| 2 | ko03010 | Ribosome | 123 | 125 | 44 | ko00561 | Glycerolipid metabolism | 22 | 25 |
| 3 | ko01230 | Biosynthesis of amino acids | 119 | 126 | 45 | ko00900 | Terpenoid backbone biosynthesis | 22 | 23 |
| 4 | ko03040 | Spliceosome | 107 | 110 | 46 | ko00510 | N-Glycan biosynthesis | 21 | 22 |
| 5 | ko00230 | Purine metabolism | 99 | 115 | 47 | ko00400 | Phenylalanine, tyrosine and tryptophan biosynthesis | 21 | 21 |
| 6 | ko03013 | RNA transport | 92 | 95 | 48 | ko00920 | Sulfur metabolism | 21 | 21 |
| 7 | ko04141 | Protein processing in endoplasmic reticulum | 77 | 82 | 49 | ko00061 | Fatty acid biosynthesis | 20 | 22 |
| 8 | ko00240 | Pyrimidine metabolism | 76 | 82 | 50 | ko00280 | Valine, leucine and isoleucine degradation | 20 | 21 |
| 9 | ko00190 | Oxidative phosphorylation | 66 | 69 | 51 | ko00220 | Arginine biosynthesis | 20 | 21 |
| 10 | ko03008 | Ribosome biogenesis in eukaryotes | 57 | 61 | 52 | ko03022 | Basal transcription factors | 19 | 21 |
| 11 | ko04120 | Ubiquitin mediated proteolysis | 55 | 64 | 53 | ko00770 | Pantothenate and CoA biosynthesis | 18 | 20 |
| 12 | ko03018 | RNA degradation | 54 | 61 | 54 | ko03430 | Mismatch repair | 17 | 22 |
| 13 | ko04144 | Endocytosis | 52 | 54 | 55 | ko00640 | Propanoate metabolism | 17 | 19 |
| 14 | ko04146 | Peroxisome | 45 | 50 | 56 | ko00130 | Ubiquinone and other terpenoid-quinone biosynthesis | 17 | 19 |
| 15 | ko03015 | mRNA surveillance pathway | 45 | 47 | 57 | ko00760 | Nicotinate and nicotinamide metabolism | 17 | 19 |
| 16 | ko00010 | Glycolysis / Gluconeogenesis | 44 | 49 | 58 | ko00053 | Ascorbate and aldarate metabolism | 17 | 18 |
| 17 | ko00620 | Pyruvate metabolism | 43 | 49 | 59 | ko00071 | Fatty acid degradation | 17 | 17 |
| 18 | ko00270 | Cysteine and methionine metabolism | 42 | 43 | 60 | ko03410 | Base excision repair | 16 | 20 |
| 19 | ko00970 | Aminoacyl-tRNA biosynthesis | 41 | 45 | 61 | ko00600 | Sphingolipid metabolism | 16 | 18 |
| 20 | ko00860 | Porphyrin and chlorophyll metabolism | 39 | 42 | 62 | ko01040 | Biosynthesis of unsaturated fatty acids | 16 | 16 |
| 21 | ko00500 | Starch and sucrose metabolism | 37 | 45 | 63 | ko03440 | Homologous recombination | 15 | 22 |
| 22 | ko00710 | Carbon fixation in photosynthetic organisms | 36 | 40 | 64 | ko00350 | Tyrosine metabolism | 14 | 16 |
| 23 | ko01212 | Fatty acid metabolism | 36 | 38 | 65 | ko00450 | Selenocompound metabolism | 14 | 16 |
| 24 | ko00520 | Amino sugar and nucleotide sugar metabolism | 35 | 37 | 66 | ko04130 | SNARE interactions in vesicular transport | 14 | 15 |
| 25 | ko04070 | Phosphatidylinositol signaling system | 35 | 36 | 67 | ko00670 | One carbon pool by folate | 14 | 14 |
| 26 | ko00260 | Glycine, serine and threonine metabolism | 34 | 39 | 68 | ko00051 | Fructose and mannose metabolism | 13 | 19 |
| 27 | ko00630 | Glyoxylate and dicarboxylate metabolism | 34 | 37 | 69 | ko02010 | ABC transporters | 13 | 16 |
| 28 | ko03050 | Proteasome | 34 | 34 | 70 | ko00906 | Carotenoid biosynthesis | 13 | 15 |
| 29 | ko00195 | Photosynthesis | 33 | 35 | 71 | ko00340 | Histidine metabolism | 13 | 14 |
| 30 | ko00330 | Arginine and proline metabolism | 32 | 36 | 72 | ko00052 | Galactose metabolism | 12 | 16 |
| 31 | ko00020 | Citrate cycle (TCA cycle) | 32 | 33 | 73 | ko00590 | Arachidonic acid metabolism | 12 | 12 |
| 32 | ko00480 | Glutathione metabolism | 32 | 32 | 74 | ko00290 | Valine, leucine and isoleucine biosynthesis | 12 | 12 |
| 33 | ko00564 | Glycerophospholipid metabolism | 31 | 38 | 75 | ko04140 | Regulation of autophagy | 11 | 13 |
| 34 | ko03420 | Nucleotide excision repair | 31 | 37 | 76 | ko00360 | Phenylalanine metabolism | 11 | 12 |
| 35 | ko01210 | 2-Oxocarboxylic acid metabolism | 30 | 30 | 77 | ko00100 | Steroid biosynthesis | 11 | 11 |
| 36 | ko04145 | Phagosome | 29 | 30 | 78 | ko04075 | Plant hormone signal transduction | 11 | 11 |
| 37 | ko03060 | Protein export | 28 | 29 | 79 | ko00410 | beta-Alanine metabolism | 10 | 12 |
| 38 | ko03030 | DNA replication | 27 | 33 | 80 | ko00592 | alpha-Linolenic acid metabolism | 10 | 11 |
| 39 | ko00250 | Alanine, aspartate and glutamate metabolism | 26 | 27 | 81 | ko00040 | Pentose and glucuronate interconversions | 10 | 11 |
| 40 | ko03020 | RNA polymerase | 26 | 26 | 82 | ko00300 | Lysine biosynthesis | 10 | 10 |
| 41 | ko00030 | Pentose phosphate pathway | 24 | 26 |  |  |  |  |  |
| 42 | ko00562 | Inositol phosphate metabolism | 23 | 24 | Total number of genes | | | 2710 | 2937 |

**Data in Brief 4**. KEGG pathways defined for *C. reinhardtii* (<http://www.kegg.jp/kegg/>). Names of the pathways (ko indexes), number of genes contained in the pathway and number of genes of the pathway detected in our transcriptomic data are mentioned.

|  | **balance state – all 16 samples together** | | | | |
| --- | --- | --- | --- | --- | --- |
|  | Identifier | Name | G_i0_ value | Definition | Description |
| **1** | **Cre03.g207050.v5.5** | **RPL29** | **-0.0441087** | **Ribosomal protein L29, component of cytosolic 80S ribosome and 60S large subunit** | **Cytosolic 80S ribosomal protein L29; Cytosolic 60S large ribosomal subunit protein L29** |
| **2** | **Cre10.g420750.v5.5** | **RPL30** | **-0.0417273** | **Ribosomal protein L3-0. component of cytosolic 80S ribosome and 60S large subunit** | **Cytosolic 80S ribosomal protein L30; Cytosolic 60S large ribosomal subunit protein L30** |
| **3** | **Cre12.g529400.v5.5** | **RPS27e1** | **-0.0412797** | **Ribosomal protein S27e isoform 1, component of 80S ribosome and 40S small subunit** | **Cytosolic 80S ribosomal protein S27e, isoform 1; Cytosolic 40S small ribosomal subunit protein S27e, isoform 1. Previously annoated as RPS27-A** |
| **4** | **Cre16.g666301.v5.5** | **RPS30** | **-0.0401102** | **Ribosomal protein S3-0. component of cytosolic 80S ribosome and 40S small subunit** | **Cytosolic 80S ribosomal protein S30; Cytosolic 40S small ribosomal subunit protein S30** |
| **5** | **Cre06.g310700.v5.5** | **RPL36a** | **-0.0400216** | **Ribosomal protein L36a, component of cytosolic 80S ribosome and 60S large subunit** | **This gene has also been annotated as RPL41 (according to the nomenclature used for the yeast ribosomal subunits) and shown to be the site of the cycloheximide resistance mutation ACT2. See PMID: 11254126. Cytosolic 80S ribosomal protein L36a; Cytosolic** |
| **6** | **Cre03.g203450.v5.5** | **RPS21** | **-0.0394877** | **Ribosomal protein S21, component of cytosolic 80S ribosome and 40S small subunit** | **Cytosolic 80S ribosomal protein S21; Cytosolic 40S small ribosomal subunit protein S21** |
| **7** | **Cre06.g282500.v5.5** | **RPL23a** | **-0.0394199** | **Ribosomal protein L23a, component of cytosolic 80S ribosome and 60S large subunit** | **Cytosolic 80S ribosomal protein L23a; Cytosolic 60S large ribosomal subunit protein L23a** |
| **8** | **Cre08.g382500.v5.5** | **RPS25** | **-0.0394116** | **Ribosomal protein S25, component of cytosolic 80S ribosome and 40S small subunit** | **Cytosolic 80S ribosomal protein S25; Cytosolic 40S small ribosomal subunit protein S25** |
| **9** | **Cre08.g360900.v5.5** | **RPS15** | **-0.039343** | **Ribosomal protein S15, component of cytosolic 80S ribosome and 40S small subunit** | **Cytosolic 80S ribosomal protein S15; Cytosolic 40S small ribosomal subunit protein S15** |
| **10** | **Cre16.g682300.v5.5** | **RPS26** | **-0.0389401** | **Ribosomal protein S26, component of cytosolic 80S ribosome and 40S small subunit** | **Cytosolic 80S ribosomal protein S26; Cytosolic 40S small ribosomal subunit protein S26** |
| **11** | **Cre02.g143050.v5.5** | **RPP2** | **-0.0388384** | **Acidic ribosomal protein P2** | **Cytosolic 80S ribosomal protein P2; Cytosolic 60S large ribosomal subunit protein P2** |
| 12 | Cre01.g066917.v5.5 | LHCBM1 | -0.0383745 | Chlorophyll a/b binding protein of LHCII | Chlorophyll a-b binding protein of LHCII |
| 13 | Cre02.g120150.v5.5 | RBCS2 | -0.0383725 | Ribulose-1,5-bisphosphate carboxylase/oxygenase small subunit 2 | RuBisCO small subunit 2, chloroplast precursor [PMID: 3820291] |
| **14** | **Cre12.g514500.v5.5** | **RPS11** | **-0.0383228** | **Ribosomal protein S11, component of cytosolic 80S ribosome and 40S small subunit** | **Cytosolic 80S ribosomal protein S11; Cytosolic 40S small ribosomal subunit protein S11** |
| 15 | Cre12.g489153.v5.5 | #N/A | -0.0381789 | #N/A | #N/A |
| **16** | **Cre07.g325746.v5.5** | **RPL38** | **-0.0380879** | **Ribosomal protein L38, component of cytosolic 80S ribosome and 60S large subunit** | **Cytosolic 80S ribosomal protein L38; Cytosolic 60S large ribosomal subunit protein L38** |
| **17** | **Cre08.g359750.v5.5** | **RPS9** | **-0.0379511** | **Ribosomal protein S9, component of cytosolic 80S ribosome and 40S small subunit** | **Cytosolic 80S ribosomal protein S9; Cytosolic 40S small ribosomal subunit protein S9** |
| **18** | **Cre10.g430400.v5.5** | **RPL37** | **-0.037616** | **Ribosomal protein L37, component of cytosolic 80S ribosome and 60S large subunit** | **Cytosolic 80S ribosomal protein L37; Cytosolic 60S large ribosomal subunit protein L37** |
| **19** | **Cre12.g494050.v5.5** | **RPL9** | **-0.037489** | **Ribosomal protein L9, component of cytosolic 80S ribosome and 60S large subunit** | **Cytosolic 80S ribosomal protein L9; Cytosolic 60S large ribosomal subunit protein L9** |
| **20** | **Cre09.g388200.v5.5** | **RPL10** | **-0.0373316** | **Ribosomal protein L1-0. component of cytosolic 80S ribosome and 60S large subunit** | **Cytosolic 80S ribosomal protein L10; Cytosolic 60S large ribosomal subunit protein L10** |
| **21** | **Cre10.g456200.v5.5** | **RPS24** | **-0.0371878** | **Ribosomal protein S24, component of cytosolic 80S ribosome and 40S small subunit** | **Cytosolic 80S ribosomal protein S24; Cytosolic 40S small ribosomal subunit protein S24. Possible locus for PR1 (paromomycin resistance) genetic marker** |
| **22** | **Cre04.g211800.v5.5** | **RPL23** | **-0.0371363** | **Ribosomal protein L23, component of cytosolic 80S ribosome and 60S large subunit** | **Cytosolic 80S ribosomal protein L23; Cytosolic 60S large ribosomal subunit protein L23** |
| **23** | **Cre12.g498250.v5.5** | **RPS17** | **-0.0370797** | **Ribosomal protein S17, component of cytosolic 80S ribosome and 40S small subunit** | **Cytosolic 80S ribosomal protein S17; Cytosolic 40S small ribosomal subunit protein S17** |
| 24 | Cre04.g214503.v5.5 | #N/A | -0.0370682 | #N/A | #N/A |
| **24** | **Cre06.g290950.v5.5** | **RPS5** | **-0.036391** | **Ribosomal protein S5, component of cytosolic 80S ribosome and 40S small subunit** | **Cytosolic 80S ribosomal protein S5; Cytosolic 40S small ribosomal subunit protein S5** |
| 26 | Cre03.g182551.v5.5 | PCY1 | -0.0362985 | Pre-apoplastocyanin | Pre-apoplastocyanin copper binding protein, PETE [PMID: 2165059; PMID: 8940133]; structure of plastocyanin PDB: 2PLT; mutant = ac208 [PMID: 8463310] |
| **27** | **Cre12.g537800.v5.5** | **RPL7** | **-0.0360244** | **Ribosomal protein L7, component of cytosolic 80S ribosome and 60S large subunit** | **Cytosolic 80S ribosomal protein L7; Cytosolic 60S large ribosomal subunit protein L7** |
| 28 | Cre14.g626700.v5.5 | PETF | -0.0360156 | Ferredoxin | 2Fe-2S containing redox protein involved in photosynthetic electron transfer, chloroplast localization [PMID: 16656453] |
| **29** | **Cre13.g568900.v5.5** | **RPL17** | **-0.0358546** | **Ribosomal protein L17, component of cytosolic 80S ribosome and 60S large subunit** | **Cytosolic 80S ribosomal protein L17; Cytosolic 60S large ribosomal subunit protein L17** |
| **30** | **Cre08.g358556.v5.5** | **RPS29** | **-0.0357983** | **Ribosomal protein S29, component of cytosolic 80S ribosome and 40S small subunit** | **Cytosolic 80S ribosomal protein S29; Cytosolic 40S small ribosomal subunit protein S29** |
| **31** | **Cre06.g272800.v5.5** | **RPS8** | **-0.0357831** | **Ribosomal protein S8, component of cytosolic 80S ribosome and 40S small subunit** | **Cytosolic 80S ribosomal protein S8; Cytosolic 40S small ribosomal subunit protein S8** |
| **32** | **Cre06.g273600.v5.5** | **RPS27a** | **-0.0357815** | **Ribosomal protein S27a, component of cytosolic 80S ribosome and 40S small subunit** | **Cytosolic 80S ribosomal protein S27a; Cytosolic 40S small ribosomal subunit protein S27a; ubiquitin region ends with amino acid residue 74. Has homology to ubiquitin superfamily** |
| **33** | **Cre12.g512600.v5.5** | **RPL18** | **-0.0356463** | **Ribosomal protein L18, component of cytosolic 80S ribosome and 60S large subunit** | **Cytosolic 80S ribosomal protein L18; Cytosolic 60S large ribosomal subunit protein L18** |
| **34** | **Cre10.g459250.v5.5** | **RPL35a** | **-0.0354754** | **Ribosomal protein L35a, component of cytosolic 80S ribosome and 60S large subunit** | **Cytosolic 80S ribosomal protein L35a; Cytosolic 60S large ribosomal subunit protein L35a** |
| **35** | **Cre17.g738300.v5.5** | **RPP1** | **-0.0354434** | **Acidic ribosomal protein P1** | **Cytosolic 80S ribosomal protein P1; Cytosolic 60S large ribosomal subunit protein P1** |
| 36 | Cre10.g420350.v5.5 | PSAE | -0.0353805 | Photosystem I 8.1 kDa reaction center subunit IV | Photosystem I reaction center subunit IV, chloroplast precursor (PSI-E) (Photosystem I 8.1 kDa protein) [PMID: 2693938] |
| **37** | **Cre01.g027000.v5.5** | **RPL11** | **-0.0353561** | **Ribosomal protein L11, component of cytosolic 80S ribosome and 60S large subunit** | **Cytosolic 80S ribosomal protein L11; Cytosolic 60S large ribosomal subunit protein L11** |
| **38** | **Cre17.g701650.v5.5** | **RPL27** | **-0.0352832** | **Ribosomal protein L27, component of cytosolic 80S ribosome and 60S large subunit** | **Cytosolic 80S ribosomal protein L27; Cytosolic 60S large ribosomal subunit protein L27** |
| **39** | **Cre14.g617900.v5.5** | **RPL35** | **-0.0352725** | **Ribosomal protein L35, component of cytosolic 80S ribosome and 60S large subunit** | **Cytosolic 80S ribosomal protein L35; Cytosolic 60S large ribosomal subunit protein L35** |
| **40** | **Cre02.g106600.v5.5** | **RPS19** | **-0.0352276** | **Ribosomal protein S19, component of cytosolic 80S ribosome and 40S small subunit** | **Cytosolic 80S ribosomal protein S19; Cytosolic 40S small ribosomal subunit protein S19** |
| **41** | **Cre12.g532550.v5.5** | **RPL13a** | **-0.0352177** | **Ribosomal protein L13a, component of cytosolic 80S ribosome and 60S large subunit** | **Cytosolic 80S ribosomal protein L13a; Cytosolic 60S large ribosomal subunit protein L13a** |
| **42** | **Cre02.g102250.v5.5** | **RPS3** | **-0.0351398** | **Ribosomal protein S3, component of cytosolic 80S ribosome and 40S small subunit** | **Cytosolic 80S ribosomal protein S3; Cytosolic 40S small ribosomal subunit protein S3** |
| **43** | **Cre06.g257150.v5.5** | **RPL37a** | **-0.035103** | **Ribosomal protein L37a, component of cytosolic 80S ribosome and 60S large subunit** | **Cytosolic 80S ribosomal protein L37a; Cytosolic 60S large ribosomal subunit protein L37a** |
| **44** | **Cre12.g498900.v5.5** | **RPS7** | **-0.0350799** | **Ribosomal protein S7, component of cytosolic 80S ribosome and 40S small subunit** | **Cytosolic 80S ribosomal protein S7; Cytosolic 40S small ribosomal subunit protein S7** |
| **45** | **Cre12.g528750.v5.5** | **RPL12** | **-0.0350349** | **Ribosomal protein L12, component of cytosolic 80S ribosome and 60S large subunit** | **Cytosolic 80S ribosomal protein L12; Cytosolic 60S large ribosomal subunit protein L12** |
| **46** | **Cre07.g357850.v5.5** | **RPL22** | **-0.034811** | **Ribosomal protein L22, component of cytosolic 80S ribosome and 60S large subunit** | **Cytosolic 80S ribosomal protein L22; Cytosolic 60S large ribosomal subunit protein L22** |
| **47** | **Cre12.g504200.v5.5** | **RPS23** | **-0.0344687** | **Ribosomal protein S23, component of cytosolic 80S ribosome and 40S small subunit** | **Cytosolic 80S ribosomal protein S23; Cytosolic 40S small ribosomal subunit protein S23** |
| 48 | Cre09.g405106.v5.5 | #N/A | -0.0343005 | #N/A | #N/A |
| **49** | **Cre12.g484050.v5.5** | **RPL36** | **-0.0342738** | **Ribosomal protein L36, component of cytosolic 80S ribosome and 60S large subunit** | **Cytosolic 80S ribosomal protein L36; Cytosolic 60S large ribosomal subunit protein L36** |
| **50** | **Cre09.g391097.v5.5** | **RPL24** | **-0.0342292** | **Ribosomal protein L24, component of cytosolic 80S ribosome and 60S large subunit** | **Cytosolic 80S ribosomal protein L24; Cytosolic 60S large ribosomal subunit protein L24** |
| 51 | Cre01.g007051.v5.5 | #N/A | -0.0341731 | #N/A | #N/A |
| 52 | Cre09.g402219.v5.5 | LCI3 | -0.0341695 | Low-CO2-inducible protein | Regulated by CCM1 [PMID: 15235119]. Acclimation to changing CO2 concentrations and light intensities was studied by Yamano et al. 2008 [PMID: 18322145]. |
| **53** | **Cre14.g621450.v5.5** | **RPL5** | **-0.0337335** | **Ribosomal protein L5, component of cytosolic 80S ribosome and 60S large subunit** | **Cytosolic 80S ribosomal protein L5; Cytosolic 60S large ribosomal subunit protein L5** |
| 54 | Cre12.g498600.v5.5 | #N/A | -0.0337325 | Eukaryotic translation elongation factor 1 alpha | Flagellar Associated Protein, found in the flagellar proteome [PMID: 15998802]. Previously annotated as just EEF1 |
| **55** | **Cre17.g701200.v5.5** | **RPL14** | **-0.0336314** | **Ribosomal protein L14, component of cytosolic 80S ribosome and 60S large subunit** | **Cytosolic 80S ribosomal protein L14; Cytosolic 60S large ribosomal subunit protein L14** |
| **56** | **Cre13.g568650.v5.5** | **RPS3a** | **-0.0334977** | **Ribosomal protein S3a, component of cytosolic 80S ribosome and 40S small subunit** | **Cytosolic 80S ribosomal protein S3a; Cytosolic 40S small ribosomal subunit protein S3a** |
| **57** | **Cre01.g047750.v5.5** | **RPL18a** | **-0.0334572** | **Ribosomal protein L18a, component of cytosolic 80S ribosome and 60S large subunit** | **Cytosolic 80S ribosomal protein L18a; Cytosolic 60S large ribosomal subunit protein L18a** |
| **58** | **Cre06.g278135.v5.5** | **RPL21** | **-0.0331582** | **Ribosomal protein L21, component of cytosolic 80S ribosome and 60S large subunit** | **Cytosolic 80S ribosomal protein L21; Cytosolic 60S large ribosomal subunit protein L21** |
| **59** | **Cre02.g101350.v5.5** | **RPL10a** | **-0.0331156** | **Ribosomal protein L10a, component of cytosolic 80S ribosome and 60S large subunit** | **Cytosolic 80S ribosomal protein L10a; Cytosolic 60S large ribosomal subunit protein L10a** |
| **60** | **Cre02.g115200.v5.5** | **RPL27a** | **-0.033038** | **Ribosomal protein L27a, component of cytosolic 80S ribosome and 60S large subunit** | **Cytosolic 80S ribosomal protein L27a; Cytosolic 60S large ribosomal subunit protein L27a. Candidate gene for the cycloheximide resistance mutation act1.** |
| 61 | Cre12.g546150.v5.5 | PETM | -0.0329136 | Cytochrome b6f complex PetM subunit | Cytochrome b6f complex chain PetM, chloroplast precursor; GI:2493687; PMID: 8631873, PMID: 8616155, PMID: 7493968 |
| 62 | Cre06.g278222.v5.5 | RCK1 | -0.0327044 | Receptor of activated protein kinase C | Receptor of activated protein kinase C 1, component of 40S small ribosomal subunit; Also cytosolic 40S small ribosomal subunit protein RACK1. Previously annotated as RACK1 and CBLP. Initially described [PMID: 2116589] as CBLP. Smith and Lee 2008 [PMID: |
| 63 | Cre12.g560950.v5.5 | PSAG | -0.0325305 | Photosystem I reaction center subunit V | (PSI-G) (P35 protein) [PMID: 2693938] |
| **64** | **Cre10.g417700.v5.5** | **RPL3** | **-0.0325262** | **Ribosomal protein L3, component of cytosolic 80S ribosome and 60S large subunit** | **Cytosolic 80S ribosomal protein L3; Cytosolic 60S large ribosomal subunit protein L3** |
| **65** | **Cre02.g075700.v5.5** | **RPL19** | **-0.0325095** | **Ribosomal protein L19, component of cytosolic 80S ribosome and 60S large subunit** | **Cytosolic 80S ribosomal protein L19; Cytosolic 60S large ribosomal subunit protein L19** |
| 66 | Cre12.g486300.v5.5 | PSAL | -0.0324252 | Photosystem I reaction center subunit XI | #N/A |
| 67 | Cre12.g529651.v5.5 | #N/A | -0.0324189 | #N/A | #N/A |
| 68 | Cre11.g467578.v5.5 | #N/A | -0.0322713 | #N/A | #N/A |
| **69** | **Cre07.g331900.v5.5** | **RPS13** | **-0.032224** | **Ribosomal protein S13, component of cytosolic 80S ribosome and 40S small subunit** | **Cytosolic 80S ribosomal protein S13; Cytosolic 40S small ribosomal subunit protein S13** |
| **70** | **Cre06.g272950.v5.5** | **RPS18** | **-0.0321851** | **Ribosomal protein S18, component of cytosolic 80S ribosome and 40S small subunit** | **Cytosolic 80S ribosomal protein S18; Cytosolic 40S small ribosomal subunit protein S18** |
| **71** | **Cre09.g411100.v5.5** | **RPS10** | **-0.032052** | **Ribosomal protein S1-0. component of cytosolic 80S ribosome and 40S small subunit** | **Cytosolic 80S ribosomal protein S10; Cytosolic 40S small ribosomal subunit protein S10** |
| **72** | **Cre14.g630100.v5.5** | **RPL13** | **-0.0319474** | **Ribosomal protein L13, component of cytosolic 80S ribosome and 60S large subunit** | **Cytosolic 80S ribosomal protein L13; Cytosolic 60S large ribosomal subunit protein L13** |
| 73 | Cre13.g577100.v5.5 | ACP2 | -0.0317649 | Acyl-carrier protein | Acyl-carrier protein |
| 74 | Cre12.g483850.v5.5 | #N/A | -0.0317034 | #N/A | #N/A |
| 75 | Cre06.g263450.v5.5 | #N/A | -0.0315772 | #N/A | #N/A |
| **76** | **Cre02.g091100.v5.5** | **RPL15** | **-0.031471** | **Ribosomal protein L15, component of cytosolic 80S ribosome and 60S large subunit** | **Cytosolic 80S ribosomal protein L15; Cytosolic 60S large subunit ribosomal protein L15** |
| **77** | **Cre01.g011000.v5.5** | **RPL6** | **-0.0314481** | **Ribosomal protein L6, component of cytosolic 80S ribosome and 60S large subunit** | **Cytosolic 80S ribosomal protein L6; Cytosolic 60S large ribosomal subunit protein L6** |
| 78 | Cre05.g234637.v5.5 | #N/A | -0.031324 | #N/A | #N/A |
| **79** | **Cre12.g520500.v5.5** | **RPP0** | **-0.0312926** | **Acidic ribosomal protein P-0. Ribosomal protein L10** | **Cytosolic 80S acidic ribosomal protein P0; Cytosolic 60S large ribosomal subunit protein P0. Ribosomal protein L10** |
| 80 | Cre12.g548950.v5.5 | LHCBM7 | -0.0309816 | Chlorophyll a/b binding protein of LHCII | Chlorophyll a-b binding protein of LHCII |
| **81** | **Cre06.g249250.v5.5** | **RPL7ae** | **-0.0309195** | **Ribosomal protein L7Ae** | **Cytosolic 80S ribosomal protein L7ae; Cytosolic 60S large ribosomal subunit protein L7ae** |
| **82** | **Cre16.g661050.v5.5** | **RPL34** | **-0.0307426** | **Ribosomal protein, L34e superfamily, component of cytosolic 80S ribosome and 60S large subunit** | **Cytosolic 80S ribosomal protein L34; Cytosolic 60S large ribosomal subunit protein L34. Belongs to Ribosomal_L34e superfamily** |
| 83 | Cre12.g548400.v5.5 | LHCBM2 | -0.0307266 | Light-harvesting protein of photosystem II | Encoding a light-harvesting antenna protein for PS2. This gene was reported under the name of LhcII-3 in PMID: 11522911, and the sequence has been deposited in Genbank (AB051205). Also designated as LhcbM2 in PMID: 16143838 and as Lhcbm2 in PMID: 14652691 |
| 84 | Cre12.g535851.v5.5 | #N/A | -0.0306301 | #N/A | #N/A |
| 85 | Cre16.g660150.v5.5 | #N/A | -0.0306154 | #N/A | #N/A |
| **86** | **Cre01.g040000.v5.5** | **RPL26** | **-0.0306063** | **Ribosomal protein L26, component of cytosolic 80S ribosome and 60S large subunit** | **Cytosolic 80S ribosomal protein L26; Cytosolic 60S large ribosomal subunit protein L26** |
| **87** | **Cre01.g039250.v5.5** | **RPS2** | **-0.0305367** | **Ribosomal protein S2, component of cytosolic 80S ribosome and 40S small subunit** | **Cytosolic 80S ribosomal protein S2; Cytosolic 40S small ribosomal subunit protein S2** |
| **88** | **Cre09.g397697.v5.5** | **RPL4** | **-0.0302693** | **Ribosomal protein L4, component of cytosolic 80S ribosome and 60S large subunit** | **Cytosolic 80S ribosomal protein L4; Cytosolic 60S large ribosomal subunit protein L4** |
| 89 | Cre06.g285250.v5.5 | LHCBM6 | -0.0301451 | Chloropyll a/b binding protein of LHCII type I, chloroplast precursor | Chloropyll a-b binding protein of LHCII type I, chloroplast precursor |
| 90 | Cre17.g724300.v5.5 | PSAK | -0.0301443 | Photosystem I reaction center subunit psaK | 8.4 kD subunit of photosystem I (polypeptide 37) [PMID: 2693938] |
| **91** | **Cre12.g510450.v5.5** | **RPS28** | **-0.0301316** | **Ribosomal protein S28, component of cytosolic 80S ribosome and 40S small subunit** | **Cytosolic 80S ribosomal protein S28; Cytosolic 40S small ribosomal subunit protein S28** |
| **92** | **Cre06.g308250.v5.5** | **RPS4** | **-0.0301189** | **Ribosomal protein S4, component of cytosolic 80S ribosome and 40S small subunit** | **Cytosolic 80S ribosomal protein S4; Cytosolic 40S small ribosomal subunit protein S4** |
| 93 | Cre06.g272650.v5.5 | LHCA8 | -0.0300836 | Light-harvesting protein of photosystem I | #N/A |
| **94** | **Cre09.g400650.v5.5** | **RPS6** | **-0.0299611** | **Ribosomal protein S6, component of cytosolic 80S ribosome and 40S small subunit** | **Cytosolic 80S ribosomal protein S6; Cytosolic 40S small ribosomal subunit protein S6** |
| 95 | Cre01.g010900.v5.5 | GAP3 | -0.0299284 | Glyceraldehyde-3-Phosphate Dehydrogenase | Glyceraldehyde 3-phosphate dehydrogenase A, chloroplast precursor (NADP-dependent glyceraldehyde phosphate dehydrogenase subunit A); corresponds to G3PA_CHLRE; found in the flagellar proteome [PMID: 15998802]. This enzyme is bispecific for NADP and NAD, |
| **96** | **Cre17.g734450.v5.5** | **PRPL19** | **-0.029736** | **Plastid ribosomal protein L19** | **Chloroplast ribosomal protein L19, imported to chloroplast; Chloroplast large ribosomal subunit protein L19** |
| 97 | Cre06.g261000.v5.5 | PSBR | -0.0296755 | 10 kDa photosystem II polypeptide | Similar to At1g7904-0. chloroplast-targeted |
| 98 | Cre04.g214150.v5.5 | THI4 | -0.0296257 | Thiazole biosynthetic enzyme; THI4 regulatory protein | Involved in the synthesis of the thiazole moiety of thiamine pyrophosphate. One of 2 splice variants.; Alternatively spliced variant of THI4 involved in the regulation of THI4a. Contains riboswitch THI RNA motif at positions 426525-426691. Regulatory p |
| 99 | Cre17.g702950.v5.5 | #N/A | -0.0295457 | #N/A | #N/A |
| 100 | Cre16.g650550.v5.5 | FAP103 | -0.0294053 | Flagellar Associated Protein, nucleoside diphosphate kinase-like | Flagellar Associated Protein similar to nucleoside diphosphate kinase, found in the flagellar proteome [PMID: 15998802] |

**Data in Brief 5**. List of the 100 most highly expressed genes in the balance constraint when all 16 samples are grouped together. Bold: transcripts encoding components of ribosome; underlined: transcripts encoding components of the photosynthetic machinery. *C. reinhardtii* genome version v5.5. #N/A means the function of the gene is unknown.

|  | **Light samples – constraint 1 – 100 most positive genes- *iclC* phenotype** | | | | **Light samples – constraint 1 – 100 most negative genes- *icl* phenotype** | | | |
| --- | --- | --- | --- | --- | --- | --- | --- | --- |
|  | Identifier | Name | G_i1_ value | Definition | Identifier | Name | G_i1_ value | Definition |
| 1 | Cre02.g141206.v5.5 | #N/A | 6.20E-02 | #N/A | Cre07.g321800.v5.5 | #N/A | -5.76E-02 | #N/A |
| 2 | Cre13.g577850.v5.5 | #N/A | 4.97E-02 | Peptidyl-prolyl cis-trans isomerase, FKBP-type | Cre16.g674151.v5.5 | #N/A | -5.69E-02 | #N/A |
| 3 | Cre03.g179820.v5.5 | #N/A | 4.86E-02 | #N/A | Cre06.g281600.v5.5 | LCI23 | -5.65E-02 | Low-CO2-inducible protein, septin-like |
| 4 | Cre16.g659300.v5.5 | #N/A | 4.73E-02 | Cytochrome b5 protein | Cre03.g155150.v5.5 | #N/A | -5.42E-02 | #N/A |
| 5 | Cre03.g144807.v5.5 | **MAS1** | 4.58E-02 | **Malate synthase** | Cre10.g426800.v5.5 | #N/A | -5.32E-02 | #N/A |
| 6 | Cre12.g546550.v5.5 | FEA1 | 4.03E-02 | Fe-assimilating protein | Cre10.g463850.v5.5 | #N/A | -5.28E-02 | #N/A |
| 7 | Cre06.g282800.v5.5 | **ICL1** | 3.92E-02 | **Isocitrate lyase** | Cre07.g328800.v5.5 | NSG13 | -5.15E-02 | Protein expressed during nitrogen-starved gametogenesis |
| 8 | Cre17.g702900.v5.5 | #N/A | 3.84E-02 | #N/A | Cre04.g220700.v5.5 | ALK2 | -5.12E-02 | Aurora-like kinase |
| 9 | Cre01.g032650.v5.5 | **TAL1** | 3.53E-02 | **Transaldolase** | Cre03.g207377.v5.5 | #N/A | -5.06E-02 | #N/A |
| 10 | Cre08.g364100.v5.5 | #N/A | 2.91E-02 | #N/A | Cre02.g075350.v5.5 | CNK1 | -5.05E-02 | NimA-related protein kinase |
| 11 | Cre12.g550500.v5.5 | #N/A | 2.87E-02 | #N/A | Cre12.g528650.v5.5 | #N/A | -5.01E-02 | #N/A |
| 12 | Cre07.g353450.v5.5 | **ACS3** | 2.87E-02 | **Acetyl-CoA synthetase/ligase** | Cre10.g464100.v5.5 | #N/A | -4.99E-02 | #N/A |
| 13 | Cre14.g630000.v5.5 | KIR2 | 2.84E-02 | Ketoacid isomerase-like protein | Cre14.g619450.v5.5 | #N/A | -4.99E-02 | #N/A |
| 14 | Cre16.g669600.v5.5 | #N/A | 2.76E-02 | #N/A | Cre06.g278161.v5.5 | #N/A | -4.95E-02 | #N/A |
| 15 | Cre10.g428100.v5.5 | #N/A | 2.76E-02 | #N/A | Cre06.g251750.v5.5 | #N/A | -4.93E-02 | #N/A |
| 16 | Cre02.g141166.v5.5 | #N/A | 2.68E-02 | #N/A | Cre09.g395251.v5.5 | #N/A | -4.90E-02 | #N/A |
| 17 | Cre12.g540500.v5.5 | #N/A | 2.47E-02 | #N/A | Cre09.g396772.v5.5 | #N/A | -4.89E-02 | #N/A |
| 18 | Cre02.g094250.v5.5 | #N/A | 2.43E-02 | #N/A | Cre12.g519850.v5.5 | #N/A | -4.88E-02 | #N/A |
| 19 | Cre14.g623176.v5.5 | #N/A | 2.38E-02 | #N/A | Cre14.g611250.v5.5 | #N/A | -4.85E-02 | #N/A |
| 20 | Cre12.g523900.v5.5 | #N/A | 2.37E-02 | #N/A | **Cre14.g622850.v5.5** | **#N/A** | **-4.84E-02** | **DNA recombination protein** |
| 21 | Cre06.g263300.v5.5 | #N/A | 2.37E-02 | #N/A | Cre11.g477350.v5.5 | #N/A | -4.79E-02 | #N/A |
| 22 | Cre02.g141400.v5.5 | **PCK1** | 2.32E-02 | **Phosphoenolpyruvate carboxykinase** | Cre02.g098300.v5.5 | #N/A | -4.78E-02 | #N/A |
| 23 | Cre01.g044450.v5.5 | #N/A | 2.31E-02 | #N/A | Cre05.g237150.v5.5 | #N/A | -4,77E-02 | #N/A |
| 24 | Cre12.g543400.v5.5 | FDH1 | 2.27E-02 | Formaldehyde dehydrogenase | Cre06.g249050.v5.5 | #N/A | -4.77E-02 | #N/A |
| 24 | Cre09.g387726.v5.5 | **AST1** | 2.23E-02 | **Aspartate aminotransferase** | Cre17.g723350.v5.5 | SUL2 | -4.75E-02 | Sulfate anion transporter |
| 26 | Cre13.g573000.v5.5 | #N/A | 2.19E-02 | #N/A | Cre03.g155100.v5.5 | #N/A | -4.74E-02 | #N/A |
| 27 | Cre07.g315400.v5.5 | #N/A | 2.17E-02 | Simlar to nitric oxide associated protein protein of CSG family | Cre03.g179450.v5.5 | SRR7 | -4.72E-02 | Scavenger receptor cysteine rich (SRCR) protein |
| 28 | Cre12.g541250.v5.5 | NAR1.5 | 2.15E-02 | Nitrite transporter | Cre07.g321750.v5.5 | #N/A | -4.71E-02 | #N/A |
| 29 | Cre15.g641200.v5.5 | #N/A | 2.14E-02 | #N/A | Cre12.g535300.v5.5 | #N/A | -4.71E-02 | #N/A |
| 30 | Cre08.g358558.v5.5 | #N/A | 2.13E-02 | #N/A | Cre10.g448977.v5.5 | #N/A | -4.70E-02 | #N/A |
| 31 | Cre50.g761397.v5.5 | #N/A | 2.13E-02 | #N/A | Cre16.g693200.v5.5 | #N/A | -4.69E-02 | #N/A |
| 32 | Cre03.g149100.v5.5 | **CIS2** | 2.12E-02 | **Citrate synthase** | Cre06.g278212.v5.5 | CGL46 | -4.69E-02 | Predicted protein |
| 33 | Cre04.g214500.v5.5 | **IDH3** | 2.09E-02 | **Isocitrate dehydrogenase, NADP-dependent** | Cre12.g500250.v5.5 | #N/A | -4.68E-02 | #N/A |
| 34 | Cre17.g731900.v5.5 | #N/A | 2.07E-02 | #N/A | **Cre03.g199450.v5.5** | **MINE2** | **-4.67E-02** | **Chloroplast division site determinant** |
| 35 | Cre02.g108350.v5.5 | #N/A | 2.02E-02 | #N/A | Cre16.g658600.v5.5 | #N/A | -4.67E-02 | #N/A |
| 36 | Cre17.g702950.v5.5 | #N/A | 1.96E-02 | #N/A | Cre16.g693450.v5.5 | FAP40 | -4.65E-02 | Flagellar Associated Protein |
| 37 | Cre07.g332800.v5.5 | #N/A | 1.93E-02 | #N/A | Cre13.g569400.v5.5 | #N/A | -4.64E-02 | #N/A |
| 38 | Cre14.g626000.v5.5 | #N/A | 1.92E-02 | #N/A | Cre01.g050700.v5.5 | #N/A | -4.64E-02 | #N/A |
| 39 | Cre03.g153400.v5.5 | #N/A | 1.88E-02 | #N/A | Cre09.g402350.v5.5 | #N/A | -4.61E-02 | #N/A |
| 40 | Cre13.g563500.v5.5 | #N/A | 1.88E-02 | #N/A | Cre03.g174400.v5.5 | CDO1 | -4.60E-02 | Cysteine dioxygenase |
| 41 | Cre07.g346650.v5.5 | #N/A | 1.85E-02 | #N/A | Cre12.g551700.v5.5 | #N/A | -4.59E-02 | #N/A |
| 42 | Cre07.g328075.v5.5 | #N/A | 1.83E-02 | #N/A | Cre04.g226600.v5.5 | #N/A | -4.57E-02 | #N/A |
| 43 | Cre03.g198850.v5.5 | #N/A | 1.81E-02 | #N/A | Cre13.g570450.v5.5 | #N/A | -4.56E-02 | #N/A |
| 44 | Cre03.g153450.v5.5 | #N/A | 1.80E-02 | #N/A | Cre12.g491050.v5.5 | RIR2 | -4.55E-02 | Ribonucleoside-diphosphate reductase R2 subunit |
| 45 | Cre06.g278136.v5.5 | #N/A | 1.77E-02 | #N/A | Cre02.g145950.v5.5 | #N/A | -4.54E-02 | #N/A |
| 46 | Cre12.g523550.v5.5 | #N/A | 1.76E-02 | #N/A | Cre09.g394065.v5.5 | #N/A | -4.53E-02 | #N/A |
| 47 | Cre01.g003200.v5.5 | #N/A | 1.73E-02 | #N/A | Cre13.g569450.v5.5 | #N/A | -4.52E-02 | #N/A |
| 48 | Cre09.g411300.v5.5 | #N/A | 1.72E-02 | #N/A | Cre06.g266550.v5.5 | #N/A | -4.51E-02 | #N/A |
| 49 | Cre03.g158100.v5.5 | #N/A | 1.68E-02 | #N/A | Cre07.g342551.v5.5 | #N/A | -4.51E-02 | #N/A |
| 50 | Cre12.g541200.v5.5 | #N/A | 1.67E-02 | #N/A | Cre17.g728750.v5.5 | #N/A | -4.50E-02 | #N/A |
| 51 | Cre08.g358553.v5.5 | #N/A | 1.67E-02 | #N/A | Cre07.g344000.v5.5 | #N/A | -4.50E-02 | #N/A |
| 52 | Cre03.g164550.v5.5 | #N/A | 1.65E-02 | #N/A | Cre12.g501250.v5.5 | #N/A | -4.47E-02 | #N/A |
| 53 | Cre01.g022283.v5.5 | #N/A | 1.64E-02 | #N/A | Cre12.g551552.v5.5 | #N/A | -4.47E-02 | #N/A |
| 54 | Cre13.g591400.v5.5 | #N/A | 1.64E-02 | #N/A | Cre10.g431900.v5.5 | #N/A | -4.46E-02 | #N/A |
| 55 | Cre13.g603750.v5.5 | #N/A | 1.62E-02 | #N/A | Cre09.g387600.v5.5 | #N/A | -4.46E-02 | #N/A |
| 56 | Cre17.g705450.v5.5 | LCI26 | 1.61E-02 | Low-CO2-induced U-box protein | Cre12.g522400.v5.5 | #N/A | -4.45E-02 | #N/A |
| 57 | Cre01.g011630.v5.5 | #N/A | 1.60E-02 | #N/A | Cre06.g299250.v5.5 | #N/A | -4.45E-02 | #N/A |
| 58 | Cre01.g043450.v5.5 | #N/A | 1.60E-02 | #N/A | Cre12.g545750.v5.5 | #N/A | -4.44E-02 | #N/A |
| 59 | Cre07.g335700.v5.5 | #N/A | 1.56E-02 | #N/A | Cre09.g413114.v5.5 | #N/A | -4.44E-02 | #N/A |
| 60 | Cre16.g679781.v5.5 | #N/A | 1.55E-02 | #N/A | Cre02.g097800.v5.5 | #N/A | -4.43E-02 | ABC transporter |
| 61 | Cre06.g278215.v5.5 | #N/A | 1.53E-02 | #N/A | Cre12.g550702.v5.5 | TEF13 | -4.43E-02 | Predicted protein |
| 62 | Cre11.g479100.v5.5 | #N/A | 1.51E-02 | #N/A | Cre09.g393600.v5.5 | #N/A | -4.43E-02 | #N/A |
| 63 | Cre13.g562300.v5.5 | #N/A | 1.51E-02 | #N/A | **Cre02.g115250.v5.5** | **POC1** | **-4.43E-02** | **Centriole proteome protein** |
| 64 | Cre04.g215050.v5.5 | #N/A | 1.50E-02 | Beta-carotene hydroxylase, putative chloroplast precursor | Cre02.g089650.v5.5 | #N/A | -4.42E-02 | #N/A |
| 65 | Cre10.g421800.v5.5 | #N/A | 1.49E-02 | #N/A | Cre02.g107000.v5.5 | #N/A | -4.40E-02 | #N/A |
| 66 | Cre02.g076300.v5.5 | UPD2 | 1.49E-02 | Uroporphyrinogen decarboxylase | **Cre17.g720350.v5.5** | **MINE1** | **-4.39E-02** | **Chloroplast division site-determinant MinE** |
| 67 | Cre06.g278147.v5.5 | #N/A | 1.47E-02 | #N/A | Cre12.g561601.v5.5 | #N/A | -4.38E-02 | #N/A |
| 68 | Cre09.g393150.v5.5 | FOX1 | 1.47E-02 | Multicopper ferroxidase | Cre08.g372550.v5.5 | CDKB1 | -4.36E-02 | Plant specific cyclin dependent kinase |
| 69 | Cre03.g167250.v5.5 | #N/A | 1.47E-02 | #N/A | Cre10.g423850.v5.5 | SRR26 | -4.36E-02 | Scavenger receptor cysteine rich (SRCR) protein |
| 70 | Cre12.g495900.v5.5 | #N/A | 1.44E-02 | #N/A | Cre03.g195950.v5.5 | #N/A | -4.35E-02 | #N/A |
| 71 | Cre08.g384700.v5.5 | #N/A | 1.44E-02 | #N/A | Cre07.g341700.v5.5 | #N/A | -4.35E-02 | #N/A |
| 72 | Cre02.g116250.v5.5 | #N/A | 1.44E-02 | #N/A | Cre04.g223100.v5.5 | CAH1 | -4.34E-02 | Carbonic anhydrase |
| 73 | Cre06.g265900.v5.5 | #N/A | 1.43E-02 | #N/A | Cre03.g194600.v5.5 | #N/A | -4.34E-02 | #N/A |
| 74 | Cre14.g630859.v5.5 | #N/A | 1.42E-02 | #N/A | Cre11.g483250.v5.5 | #N/A | -4.33E-02 | #N/A |
| 75 | Cre09.g391400.v5.5 | #N/A | 1.41E-02 | #N/A | Cre07.g325742.v5.5 | #N/A | -4.32E-02 | #N/A |
| 76 | Cre12.g487150.v5.5 | #N/A | 1.41E-02 | #N/A | Cre01.g007350.v5.5 | MOT42 | -4.32E-02 | Predicted protein |
| 77 | Cre02.g119000.v5.5 | #N/A | 1.40E-02 | #N/A | Cre12.g512500.v5.5 | #N/A | -4.30E-02 | #N/A |
| 78 | Cre05.g241655.v5.5 | #N/A | 1.40E-02 | #N/A | Cre16.g672497.v5.5 | #N/A | -4.30E-02 | #N/A |
| 79 | Cre14.g633903.v5.5 | #N/A | 1.40E-02 | #N/A | Cre14.g616800.v5.5 | #N/A | -4.29E-02 | #N/A |
| 80 | Cre19.g751047.v5.5 | #N/A | 1.40E-02 | #N/A | Cre03.g207900.v5.5 | CYCA1 | -4.29E-02 | A-type cyclin |
| 81 | Cre06.g278148.v5.5 | #N/A | 1.39E-02 | #N/A | **Cre06.g295700.v5.5** | **MCM3** | **-4.29E-02** | **Minichromosome maintenance protein** |
| 82 | Cre12.g495850.v5.5 | #N/A | 1.38E-02 | #N/A | Cre09.g388100.v5.5 | #N/A | -4.28E-02 | #N/A |
| 83 | Cre02.g109600.v5.5 | #N/A | 1.36E-02 | Inositol monophosphatase | Cre02.g108150.v5.5 | #N/A | -4.28E-02 | #N/A |
| 84 | Cre14.g630847.v5.5 | #N/A | 1.36E-02 | #N/A | Cre17.g718400.v5.5 | #N/A | -4.27E-02 | #N/A |
| 85 | Cre17.g700750.v5.5 | #N/A | 1.36E-02 | #N/A | Cre03.g204450.v5.5 | #N/A | -4.27E-02 | #N/A |
| 86 | Cre14.g615450.v5.5 | #N/A | 1.36E-02 | #N/A | Cre18.g749747.v5.5 | #N/A | -4.26E-02 | Similar to Flagellar Associated Protein FAP281 |
| 87 | Cre07.g346600.v5.5 | #N/A | 1.34E-02 | #N/A | Cre10.g456350.v5.5 | #N/A | -4.26E-02 | #N/A |
| 88 | Cre50.g761447.v5.5 | #N/A | 1.34E-02 | #N/A | Cre08.g376950.v5.5 | MMP14 | -4.25E-02 | Matrix metalloproteinase |
| 89 | Cre17.g718150.v5.5 | #N/A | 1.34E-02 | #N/A | Cre17.g723400.v5.5 | #N/A | -4.24E-02 | #N/A |
| 90 | Cre17.g700950.v5.5 | FDX5 | 1.33E-02 | Apoferredoxin | Cre17.g697500.v5.5 | #N/A | -4.23E-02 | #N/A |
| 91 | Cre17.g703700.v5.5 | **SCL2** | 1.33E-02 | **Succinate-CoA ligase beta chain** | **Cre02.g104800.v5.5** | **HTV1** | **-4.23E-02** | **Histone H3 variant** |
| 92 | Cre06.g254150.v5.5 | UTP1 | 1.32E-02 | Nucleolar protein, component of the U3 processome | Cre01.g004750.v5.5 | #N/A | -4.22E-02 | #N/A |
| 93 | Cre12.g547650.v5.5 | #N/A | 1.32E-02 | #N/A | Cre12.g505300.v5.5 | #N/A | -4.21E-02 | #N/A |
| 94 | Cre12.g548950.v5.5 | LHCBM7 | 1.31E-02 | Chlorophyll a/b binding protein of LHCII | Cre16.g666300.v5.5 | #N/A | -4.21E-02 | #N/A |
| 95 | Cre02.g073850.v5.5 | CGL54 | 1.30E-02 | Predicted protein | Cre03.g170550.v5.5 | #N/A | -4.20E-02 | #N/A |
| 96 | Cre01.g020305.v5.5 | #N/A | 1.30E-02 | #N/A | Cre14.g632100.v5.5 | #N/A | -4.19E-02 | #N/A |
| 97 | Cre04.g232104.v5.5 | LHCBM3 | 1.30E-02 | Light-harvesting complex II chlorophyll a/b binding protein M3 | Cre06.g298750.v5.5 | AOT4 | -4.19E-02 | Amino acid transporter |
| 98 | Cre03.g182100.v5.5 | #N/A | 1.29E-02 | #N/A | Cre01.g053800.v5.5 | #N/A | -4.18E-02 | #N/A |
| 99 | Cre09.g387875.v5.5 | IPY3 | 1.29E-02 | Soluble inorganic pyrophosphatase | Cre06.g311700.v5.5 | #N/A | -4.16E-02 | #N/A |
| 100 | Cre07.g329767.v5.5 | #N/A | 1.28E-02 | #N/A | Cre12.g502600.v5.5 | SLT1 | -4.15E-02 | Sodium/sulfate co-transporter |

**Data in Brief 6**. List of the 100 most positive expressed genes (*iclC* phenotype) (right) and of the 100 most negative expressed genes (*icl* phenotype) (left) of the first constraint when 8 light samples are analysed separately. Red: transcripts encoding components for acetate metabolization; bold: transcripts encoding components of cell division. *C. reinhardtii* genome version v5.5. #N/A means the function of the gene is unknown.

|  | **Dark samples – constraint 2 – 100 most positive genes- *iclC* phenotype** | | | | **Dark samples – constraint 2 – 100 most negative genes- *icl* phenotype** | | | |
| --- | --- | --- | --- | --- | --- | --- | --- | --- |
|  | Identifier | Name | G_i2_ value | Definition | Identifier | Name | G_i2_ value | Definition |
| 1 | Cre02.g141206.v5.5 | #N/A | 5.24E-02 | #N/A | **Cre04.g223100.v5.5** | **CAH1** | **-9.35E-02** | **Carbonic anhydrase** |
| 2 | Cre17.g700950.v5.5 | FDX5 | 4.65E-02 | Apoferredoxin | **Cre09.g399552.v5.5** | **LCR1** | **-8.04E-02** | **Low-CO2 response regulator, Myb-like transcription factor** |
| 3 | Cre02.g141166.v5.5 | #N/A | 4.40E-02 | #N/A | Cre16.g674151.v5.5 | #N/A | -7.45E-02 | #N/A |
| 4 | Cre06.g282800.v5.5 | ICL1 | 3.88E-02 | Isocitrate lyase | Cre17.g723400.v5.5 | #N/A | -7.26E-02 | #N/A |
| 5 | Cre01.g011630.v5.5 | #N/A | 3.63E-02 | #N/A | Cre26.g756747.v5.5 | #N/A | -6.66E-02 | #N/A |
| 6 | Cre12.g546550.v5.5 | FEA1 | 3.62E-02 | Fe-assimilating protein | Cre12.g554929.v5.5 | #N/A | -6.47E-02 | #N/A |
| 7 | Cre16.g659300.v5.5 | #N/A | 3.14E-02 | Cytochrome b5 protein | *Cre17.g723350.v5.5* | *SUL2* | *-6.42E-02* | *Sulfate anion transporter* |
| 8 | Cre03.g180750.v5.5 | MES1 | 3.12E-02 | Cobalamin-independent methionine synthase | Cre16.g681351.v5.5 | #N/A | -6.27E-02 | #N/A |
| 9 | Cre08.g364100.v5.5 | #N/A | 3.10E-02 | #N/A | Cre06.g298802.v5.5 | #N/A | -6.22E-02 | #N/A |
| 10 | Cre12.g546600.v5.5 | FEA2 | 2.71E-02 | Fe-assimilating protein | Cre07.g342551.v5.5 | #N/A | -6.18E-02 | #N/A |
| 11 | Cre01.g036950.v5.5 | #N/A | 2.70E-02 | #N/A | Cre09.g392208.v5.5 | #N/A | -6.11E-02 | #N/A |
| 12 | Cre10.g417600.v5.5 | #N/A | 2.58E-02 | #N/A | *Cre02.g144700.v5.5* | *PTB5* | *-6.09E-02* | *Sodium/phosphate symporter* |
| 13 | Cre09.g389356.v5.5 | #N/A | 2.57E-02 | #N/A | Cre13.g579650.v5.5 | #N/A | -5.84E-02 | #N/A |
| 14 | Cre12.g543400.v5.5 | FDH1 | 2.51E-02 | Formaldehyde dehydrogenase | Cre07.g328800.v5.5 | NSG13 | -5.77E-02 | Protein expressed during nitrogen-starved gametogenesis |
| 15 | Cre07.g356600.v5.5 | #N/A | 2.50E-02 | #N/A | Cre05.g234661.v5.5 | BCS1 | -5.75E-02 | Ubiquinol:cytochrome c oxidoreductase biogenesis factor |
| 16 | Cre12.g541400.v5.5 | #N/A | 2.42E-02 | #N/A | *Cre12.g502600.v5.5* | *SLT1* | *-5.73E-02* | *Sodium/sulfate co-transporter* |
| 17 | Cre13.g586300.v5.5 | FKB12 | 2.36E-02 | Peptidyl-prolyl cis-trans isomerase, FKBP-type | Cre10.g447800.v5.5 | #N/A | -5.71E-02 | #N/A |
| 18 | Cre12.g555500.v5.5 | #N/A | 2.32E-02 | #N/A | Cre17.g725750.v5.5 | #N/A | -5.56E-02 | #N/A |
| **19** | **Cre01.g062172.v5.5** | **HBV1** | **2.29E-02** | **Histone H2B variant** | Cre05.g236650.v5.5 | #N/A | -5.50E-02 | #N/A |
| 20 | Cre07.g344400.v5.5 | #N/A | 2.27E-02 | #N/A | Cre03.g163950.v5.5 | CDO2 | -5.50E-02 | Cysteine dioxygenase |
| 21 | Cre02.g080450.v5.5 | #N/A | 2.22E-02 | #N/A | Cre03.g151650.v5.5 | #N/A | -5.44E-02 | #N/A |
| 22 | Cre03.g144967.v5.5 | #N/A | 2.21E-02 | #N/A | **Cre09.g394473.v5.5** | **LCI9** | **-5.43E-02** | **Low-CO2-inducible protein** |
| 23 | Cre06.g293950.v5.5 | SHMT2 | 2.19E-02 | Serine hydroxymethyltransferase 2 | Cre03.g155150.v5.5 | #N/A | -5.32E-02 | #N/A |
| 24 | Cre14.g632100.v5.5 | #N/A | 2.17E-02 | #N/A | Cre06.g293100.v5.5 | #N/A | -5.29E-02 | #N/A |
| 24 | Cre12.g520050.v5.5 | #N/A | 2.15E-02 | #N/A | Cre12.g551700.v5.5 | #N/A | -5.28E-02 | #N/A |
| **26** | **Cre09.g399911.v5.5** | **CDC20** | **2.12E-02** | **Activator and specificity subunit of anaphase promoting complex** | Cre10.g431900.v5.5 | #N/A | -5.25E-02 | #N/A |
| 27 | Cre14.g619450.v5.5 | #N/A | 2.11E-02 | #N/A | Cre06.g301750.v5.5 | #N/A | -5.23E-02 | #N/A |
| 28 | Cre13.g586000.v5.5 | #N/A | 2.07E-02 | #N/A | **Cre16.g663450.v5.5** | **#N/A** | **-5.15E-02** | **Low-CO2-inducible membrane protein** |
| 29 | Cre01.g053800.v5.5 | #N/A | 2.06E-02 | #N/A | Cre10.g429000.v5.5 | #N/A | -5.13E-02 | #N/A |
| 30 | Cre17.g708750.v5.5 | #N/A | 2.03E-02 | #N/A | Cre13.g570801.v5.5 | #N/A | -5.01E-02 | #N/A |
| 31 | Cre03.g204250.v5.5 | SAH1 | 2.03E-02 | S-Adenosyl homocysteine hydrolase | Cre11.g468050.v5.5 | #N/A | -5.00E-02 | #N/A |
| 32 | Cre02.g101700.v5.5 | #N/A | 2.02E-02 | #N/A | Cre12.g505100.v5.5 | #N/A | -4.99E-02 | #N/A |
| 33 | Cre08.g358527.v5.5 | #N/A | 2.02E-02 | #N/A | Cre09.g403550.v5.5 | #N/A | -4.97E-02 | #N/A |
| **34** | **Cre17.g726500.v5.5** | **ORC4** | **2.02E-02** | **Origin recognition complex subunit 4** | Cre07.g342552.v5.5 | #N/A | -4.96E-02 | #N/A |
| 35 | Cre02.g144750.v5.5 | PTB4 | 2.02E-02 | Sodium/phosphate symporter | Cre12.g505050.v5.5 | #N/A | -4.96E-02 | #N/A |
| 36 | Cre04.g215050.v5.5 | #N/A | 2.02E-02 | Beta-carotene hydroxylase, putative chloroplast precursor | Cre07.g333350.v5.5 | #N/A | -4.94E-02 | #N/A |
| 37 | Cre12.g523900.v5.5 | #N/A | 2.01E-02 | #N/A | Cre12.g518800.v5.5 | #N/A | -4.90E-02 | #N/A |
| 38 | Cre14.g618250.v5.5 | #N/A | 2.00E-02 | #N/A | Cre10.g428966.v5.5 | #N/A | -4.87E-02 | #N/A |
| **39** | **Cre06.g295700.v5.5** | **MCM3** | **2.00E-02** | **Minichromosome maintenance protein** | Cre06.g287350.v5.5 | #N/A | -4.86E-02 | #N/A |
| **40** | **Cre06.g259150.v5.5** | **EFG8** | **2.00E-02** | **Mitochondrial translation factor Tu** | Cre16.g662600.v5.5 | #N/A | -4.85E-02 | #N/A |
| 41 | Cre12.g495050.v5.5 | #N/A | 1.99E-02 | #N/A | Cre02.g106450.v5.5 | #N/A | -4.82E-02 | #N/A |
| 42 | Cre11.g482700.v5.5 | #N/A | 1.98E-02 | #N/A | Cre05.g239450.v5.5 | #N/A | -4.79E-02 | #N/A |
| 43 | Cre03.g205750.v5.5 | #N/A | 1.98E-02 | #N/A | Cre01.g029000.v5.5 | #N/A | -4.76E-02 | Ubiquinone/menaquinone biosynthesis methyltransferase |
| 44 | Cre12.g561601.v5.5 | #N/A | 1.95E-02 | #N/A | Cre16.g681400.v5.5 | #N/A | -4.76E-02 | #N/A |
| 45 | Cre12.g559800.v5.5 | #N/A | 1.95E-02 | #N/A | Cre12.g485600.v5.5 | #N/A | -4.75E-02 | #N/A |
| 46 | Cre16.g688190.v5.5 | #N/A | 1.95E-02 | #N/A | Cre14.g617400.v5.5 | HSP22F | -4.73E-02 | Heat shock protein 22F |
| 47 | Cre50.g761397.v5.5 | #N/A | 1.92E-02 | #N/A | Cre13.g585000.v5.5 | #N/A | -4.72E-02 | #N/A |
| 48 | Cre07.g339250.v5.5 | #N/A | 1.92E-02 | #N/A | Cre12.g495952.v5.5 | #N/A | -4.69E-02 | #N/A |
| 49 | Cre10.g441250.v5.5 | #N/A | 1.91E-02 | #N/A | Cre02.g107000.v5.5 | #N/A | -4.69E-02 | #N/A |
| 50 | Cre08.g377100.v5.5 | ADK6 | 1.90E-02 | Adenylate kinase | Cre01.g004750.v5.5 | #N/A | -4.67E-02 | #N/A |
| 51 | Cre09.g407600.v5.5 | #N/A | 1.89E-02 | #N/A | Cre06.g278107.v5.5 | #N/A | -4.67E-02 | #N/A |
| 52 | Cre12.g523300.v5.5 | GTR22 | 1.89E-02 | Glycosyltransferase | Cre05.g238311.v5.5 | #N/A | -4.63E-02 | #N/A |
| 53 | Cre19.g751047.v5.5 | #N/A | 1.87E-02 | #N/A | Cre03.g195950.v5.5 | #N/A | -4.60E-02 | #N/A |
| **54** | **Cre14.g622850.v5.5** | **#N/A** | **1.86E-02** | **DNA recombination protein** | Cre08.g367500.v5.5 | LHCSR2 | -4.60E-02 | Stress-related chlorophyll a/b binding protein 2 |
| 55 | Cre06.g261300.v5.5 | #N/A | 1.86E-02 | #N/A | Cre13.g569450.v5.5 | #N/A | -4.59E-02 | #N/A |
| 56 | Cre14.g611250.v5.5 | #N/A | 1.85E-02 | #N/A | Cre16.g686203.v5.5 | #N/A | -4.55E-02 | #N/A |
| 57 | Cre17.g702900.v5.5 | #N/A | 1.85E-02 | #N/A | Cre07.g352550.v5.5 | RDP3 | -4.50E-02 | Putative rhodanese domain phosphatase |
| 58 | Cre01.g010296.v5.5 | #N/A | 1.84E-02 | #N/A | Cre13.g569500.v5.5 | #N/A | -4.44E-02 | #N/A |
| 59 | Cre12.g551050.v5.5 | #N/A | 1.84E-02 | #N/A | Cre13.g569400.v5.5 | #N/A | -4.43E-02 | #N/A |
| 60 | Cre06.g251750.v5.5 | #N/A | 1.84E-02 | #N/A | Cre02.g144734.v5.5 | #N/A | -4.41E-02 | #N/A |
| 61 | Cre17.g719900.v5.5 | PWD1 | 1.84E-02 | Phosphoglucan water dikinase | **Cre03.g162800.v5.5** | **LCI1** | **-4.40E-02** | **Low-CO2-inducible membrane protein** |
| 62 | Cre02.g092400.v5.5 | #N/A | 1.84E-02 | #N/A | Cre13.g605500.v5.5 | #N/A | -4.35E-02 | #N/A |
| 63 | Cre13.g562475.v5.5 | #N/A | 1.83E-02 | #N/A | Cre14.g617450.v5.5 | HSP22E | -4.34E-02 | Heat shock protein 22E |
| 64 | Cre05.g248150.v5.5 | #N/A | 1.83E-02 | #N/A | Cre02.g093750.v5.5 | NRX2 | -4.34E-02 | Nucleoredoxin 2 |
| 65 | Cre01.g032650.v5.5 | TAL1 | 1.82E-02 | Transaldolase | Cre11.g477350.v5.5 | #N/A | -4.33E-02 | #N/A |
| 66 | Cre08.g365900.v5.5 | LHCSR1 | 1.82E-02 | Stress-related chlorophyll a/b binding protein 1 | Cre03.g201417.v5.5 | #N/A | -4.31E-02 | #N/A |
| 67 | Cre16.g684700.v5.5 | #N/A | 1.82E-02 | #N/A | Cre06.g310950.v5.5 | #N/A | -4.30E-02 | #N/A |
| 68 | Cre06.g261450.v5.5 | #N/A | 1.82E-02 | #N/A | Cre15.g642865.v5.5 | #N/A | -4.28E-02 | #N/A |
| 69 | Cre17.g731900.v5.5 | #N/A | 1.81E-02 | #N/A | Cre07.g343050.v5.5 | #N/A | -4.22E-02 | #N/A |
| 70 | Cre01.g024200.v5.5 | #N/A | 1,81E-02 | #N/A | Cre06.g311050.v5.5 | #N/A | -4.18E-02 | #N/A |
| 71 | Cre17.g711150.v5.5 | #N/A | 1.80E-02 | #N/A | Cre13.g583450.v5.5 | #N/A | -4.17E-02 | #N/A |
| 72 | Cre06.g278224.v5.5 | MRPL16 | 1.80E-02 | Putative mitochondrial ribosomal protein L16, imported to mitochondria | Cre04.g214750.v5.5 | #N/A | -4.14E-02 | Endosomal R-SNARE protein, VAMP-like family (R.III) |
| 73 | Cre12.g538150.v5.5 | #N/A | 1 .78E-02 | #N/A | Cre07.g355500.v5.5 | #N/A | -4.11E-02 | #N/A |
| 74 | Cre12.g498150.v5.5 | #N/A | 1.78E-02 | #N/A | *Cre02.g095076.v5.5* | *MFT10* | *-4.02E-02* | *Major facilitator superfamily transporter, involved in circadian rhythm control* |
| 75 | Cre09.g387875.v5.5 | IPY3 | 1.77E-02 | Soluble inorganic pyrophosphatase | Cre11.g467531.v5.5 | FAP15 | -3.99E-02 | Flagellar Associated Protein |
| 76 | Cre14.g630859.v5.5 | #N/A | 1.77E-02 | #N/A | *Cre06.g309000.v5.5* | *NAR1.2* | *-3.98E-02* | *Anion transporter* |
| 77 | Cre12.g550250.v5.5 | #N/A | 1.77E-02 | #N/A | *Cre06.g298750.v5.5* | *AOT4* | *-3.97E-02* | *Amino acid transporter* |
| 78 | Cre13.g566626.v5.5 | #N/A | 1.76E-02 | #N/A | Cre08.g366700.v5.5 | #N/A | -3.94E-02 | #N/A |
| 79 | Cre11.g467641.v5.5 | #N/A | 1.75E-02 | #N/A | **Cre06.g281600.v5.5** | **LCI23** | **-3.94E-02** | **Low-CO2-inducible protein, septin-like** |
| 80 | Cre01.g026450.v5.5 | #N/A | 1.75E-02 | Serine/arginine-rich pre-mRNA splicing factor | Cre13.g569150.v5.5 | #N/A | -3.94E-02 | #N/A |
| 81 | Cre16.g678150.v5.5 | #N/A | 1.75E-02 | #N/A | Cre02.g077750.v5.5 | FAP211 | -3.87E-02 | Flagellar Associated Protein |
| **82** | **Cre17.g746347.v5.5** | **UNG1** | **1.75E-02** | **Uracil DNA glycosylase** | Cre06.g278098.v5.5 | MCC1 | -3.87E-02 | Methylcrotonoyl-CoA carboxylase alpha subunit |
| **83** | **Cre03.g172550.v5.5** | **PRM1** | **1.75E-02** | **Protein-/Histone-arginine N-methyltransferase** | Cre09.g393953.v5.5 | #N/A | -3.86E-02 | #N/A |
| 84 | Cre02.g102450.v5.5 | #N/A | 1.73E-02 | #N/A | Cre08.g367400.v5.5 | LHCSR3 | -3.85E-02 | Stress-related chlorophyll a/b binding protein 3 |
| 85 | Cre06.g249050.v5.5 | #N/A | 1.73E-02 | #N/A | Cre06.g259550.v5.5 | #N/A | -3.82E-02 | #N/A |
| 86 | Cre05.g244400.v5.5 | #N/A | 1.73E-02 | #N/A | Cre12.g504900.v5.5 | #N/A | -3.82E-02 | #N/A |
| 87 | Cre14.g623176.v5.5 | #N/A | 1.73E-02 | #N/A | Cre03.g176833.v5.5 | #N/A | -3.81E-02 | #N/A |
| 88 | Cre01.g019250.v5.5 | #N/A | 1.71E-02 | Putative dTDP-glucose 4-6-dehydratase | Cre02.g095080.v5.5 | #N/A | -3.74E-02 | #N/A |
| **89** | **Cre12.g558100.v5.5** | **PRM2** | **1.71E-02** | **Protein-/Histone-arginine N-methyltransferase** | Cre16.g685000.v5.5 | #N/A | -3.74E-02 | #N/A |
| 90 | Cre06.g298600.v5.5 | #N/A | 1.70E-02 | #N/A | Cre01.g053288.v5.5 | #N/A | -3.72E-02 | #N/A |
| 91 | Cre12.g485150.v5.5 | GAP1 | 1.70E-02 | Glyceraldehyde 3-phosphate dehydrogenase | Cre11.g467693.v5.5 | #N/A | -3.72E-02 | #N/A |
| **92** | **Cre12.g515850.v5.5** | **PCN1** | **1.70E-02** | **Proliferating cell nuclear antigen homolog** | Cre12.g554400.v5.5 | #N/A | -3.70E-02 | #N/A |
| 93 | Cre03.g190800.v5.5 | #N/A | 1.70E-02 | #N/A | Cre16.g694500.v5.5 | #N/A | -3.69E-02 | DnaJ-like protein |
| 94 | Cre17.g705550.v5.5 | #N/A | 1.70E-02 | #N/A | Cre09.g396809.v5.5 | #N/A | -3.68E-02 | #N/A |
| 95 | Cre09.g403071.v5.5 | #N/A | 1.69E-02 | #N/A | Cre04.g215900.v5.5 | #N/A | -3.64E-02 | #N/A |
| 96 | Cre16.g679350.v5.5 | #N/A | 1.69E-02 | #N/A | Cre12.g516700.v5.5 | #N/A | -3.62E-02 | #N/A |
| 97 | Cre07.g318276.v5.5 | #N/A | 1.69E-02 | #N/A | Cre04.g217925.v5.5 | #N/A | -3.61E-02 | #N/A |
| 98 | Cre16.g684850.v5.5 | #N/A | 1.69E-02 | #N/A | Cre02.g093800.v5.5 | NRX3 | -3.61E-02 | Nucleoredoxin |
| 99 | Cre06.g282850.v5.5 | #N/A | 1.69E-02 | #N/A | Cre03.g144144.v5.5 | #N/A | -3.60E-02 | #N/A |
| 100 | Cre15.g635400.v5.5 | ZYS3 | 1.69E-02 | Zygote-specific protein | Cre17.g734725.v5.5 | #N/A | -3.54E-02 | #N/A |

**Data in Brief 7.** List of the 100 most positive expressed genes (*iclC* phenotype) (right) and 100 most negative genes (*icl* phenotype) (left) of the second constraint when 8 dark samples are analysed separately. Bold: transcripts encoding components of the division machinery; red: transcripts encoding enzymes related to low carbon availability; underlined: transcripts related to stressful conditions; italis; transcripts encoding transporters. *C. reinhardtii* genome version v5.5. #N/A means the function of the gene is unknown.
